# Genome-wide analysis of filamentous temperature-sensitive H protease (ftsH) gene family in soybean

**DOI:** 10.1186/s12864-024-10389-w

**Published:** 2024-05-27

**Authors:** Jiabao Wang, Lu Liu, Rui Luo, Qi Zhang, Xinyu Wang, Fenglou Ling, Piwu Wang

**Affiliations:** 1 JiLin Agricultural University, Changchun, China; 2 East China Normal University, Shanghai, China

**Keywords:** Soybean, *ftsH* gene family, Phylogenetic analysis, Expression analysis, Subcellular localization

## Abstract

**Background:**

The filamentous temperature-sensitive H protease (*ftsH*) gene family belongs to the ATP-dependent zinc metalloproteins, and *ftsH* genes play critical roles in plant chloroplast development and photosynthesis.

**Results:**

In this study, we performed genome-wide identification and a systematic analysis of soybean *ftsH* genes. A total of 18 *GmftsH* genes were identified. The subcellular localization was predicted to be mainly in cell membranes and chloroplasts, and the gene structures, conserved motifs, evolutionary relationships, and expression patterns were comprehensively analyzed. Phylogenetic analysis of the *ftsH* gene family from soybean and various other species revealed six distinct clades, all of which showed a close relationship to *Arabidopsis thaliana*. The members of the *GmftsH* gene family were distributed on 13 soybean chromosomes, with intron numbers ranging from 3 to 15, 13 pairs of repetitive segment. The covariance between these genes and related genes in different species of *Oryza sativa*, *Zea mays*, and *Arabidopsis thaliana* was further investigated. The transcript expression data revealed that the genes of this family showed different expression patterns in three parts, the root, stem, and leaf, and most of the genes were highly expressed in the leaves of the soybean plants. Fluorescence-based real-time quantitative PCR (qRT-PCR) showed that the expression level of *GmftsH* genes varied under different stress treatments. Specifically, the genes within this family exhibited various induction levels in response to stress conditions of 4℃, 20% PEG-6000, and 100 mmol/L NaCl. These findings suggest that the *GmftsH* gene family may play a crucial role in the abiotic stress response in soybeans. It was also found that the *GmftsH7* gene was localized on the cell membrane, and its expression was significantly upregulated under 4 ℃ treatment. In summary, by conducting a genome-wide analysis of the *GmftsH* gene family, a strong theoretical basis is established for future studies on the functionality of *GmftsH* genes.

**Conclusions:**

This research can potentially serve as a guide for enhancing the stress tolerance characteristics of soybean.

**Supplementary Information:**

The online version contains supplementary material available at 10.1186/s12864-024-10389-w.

## Introduction

Chloroplast proteases play a vital role in maintaining the balance of proteins and regulating the remodeling of chloroplast proteins [1, 2]. Two significant types of chloroplast proteases, Clp and filamentous temperature-sensitive H (ftsH), are involved in the formation of multi-subunit complexes consisting of different gene products. They are associated with various biological functions of chloroplasts, resistance to stress conditions, and the degradation of chloroplasts in aging leaves [3]. A protease initially discovered in a heat-sensitive mutant of *E. coli* was named ftsH [4]. Recent studies have identified and analyzed the *ftsH* gene family, recognizing its significance in a variety of organisms. This includes prokaryotes like Lactobacillus plantarum and *Escherichia coli* [5–7], as well as several plant species such as *Arabidopsis thaliana*, tobacco, rice, corn, and peanuts [8–10]. The majority of genes in this gene family are present in the nucleus and chloroplast of plant cells [11, 12].

Under intense light conditions, the D1 protein in plants that is bound to the photosystem II reaction center will be damaged, leading to photoinhibition of photosystem II. Specific proteases will remove the damaged D1 protein, an d the metalloproteinase ftsH, located on the thylakoid membrane, participates in the proteolysis process, thereby reducing the damage to photosystem II caused by intense irradiation [13]. The *ftsH* genes are keys in regulating plant chloroplast development and photosynthesis. For example, a small amount of *Arabidopsis thaliana* ftsH protein accumulates during the seedling stage. After exposure to strong light treatment, the expression levels of most *AtftsH* genes are significantly increased [14]. The leaves of *Arabidopsis thaliana ftsH* deletion mutant var2-2 are more susceptible to photoinhibition than wild-type leaves, proving that ftsH is a necessary protease for the regular operation of the photoinhibition protection mechanism in plants [15].

The structural characteristics of the ftsH protein are the basis for its complex formation [16]. Most of the ftsH protein structures of *E. coli* contain two transmembrane regions at the N-terminus [17]. Studies have found that most ftsH molecules interact, and ftsH–ftsH binding requires the N-terminal transmembrane region [18]. The complexes formed by multiple ftsH homologs play a role in maintaining the normal biological activity of cells [9]. The accumulation of two proteins, AtFtsH2 and AtFtsH5, in *Arabidopsis thaliana* is coordinated. In mutants lacking one of these proteins, there is an observed increase in the accumulation of the other protein. Experimental results have confirmed that AtFtsH2 and AtFtsH5 proteins exist in heterogeneous complexes within the plant [19].

Studies have identified and analyzed various members of the *ftsH* gene family in plants. There are 12 *AtftsHs* genes in the Arabidopsis genome, among which *AtftsH1*, *AtftsH5*, and *AtftsH2* are related to light dependence [20, 21]. In the tobacco genome, 20 *NtftsHs* genes have been identified, including 3 *NtftsH* genes each on chromosomes Nt17 and Nt24; 2 on chromosomes Nt12, Nt16, and Nt23; and one on Nt02, Nt05, Nt06, Nt08, Nt14, Nt18, Nt19, and Nt22 [22]. Kato et al. employed RNA interference (RNAi) to suppress the expression of the *ftsH* gene in tobacco. Their investigation revealed that tobacco plants with a knockout of the *ftsH* gene exhibited leaf discoloration. The extent of discoloration displayed a negative correlation with the expression level of *ftsH* gene [23]. Yue et al. isolated two genes, *ZmftsH2A* and *ZmftsH2B*, from maize. RT-PCR analysis showed that drought stress and ABA treatment significantly upregulated the expression of *ZmftsH2B* gene in maize [24].

Currently, research on functional verification of the *ftsH* gene family in plants is mainly focused on photosynthesis and abiotic stress [25, 26]. Ivashuta et al. found that in alfalfa seedlings grown under dark conditions, transcription of the *ftsH* gene was induced by low temperature, revealing the independent regulatory effects of low temperature and light on gene expression [27]. Rangan’s study found that wheat at 14 dpa under high-temperature stress in the early and late grain-filling stages triggered upregulation of the expression of genes related to ftsH protease and RuBisCO active enzymes. These proteins may be closely related to the continued grain-filling process of wheat grains [28]. Yin et al. study utilized a completely randomized design, setting up six replications to measure plant seed yield and photosynthesis-related parameters. A notable connection was observed between the expression level of the gene and the temporary parameters of chlorophyll fluorescence, suggesting that *GmftsH9* might play a role in controlling PSII function [29].

In this genome-level study, we utilized bioinformatics techniques to identify *GmftsH* family genes. We investigated various aspects, such as the physical and chemical properties, gene structure, evolution, conserved structure, intraspecific collinearity, and tissue expression of *GmftsH* gene family members. The objective was to establish a theoretical basis for further understanding the involvement of *GmftsH* genes in soybean’s growth, development, and ability to withstand abiotic stress.

## Materials and methods

### Identification of GmftsH gene family

This study’s genome sequences, genome annotation files, and amino acid sequences were obtained from the Phytozome 13 plant genome database (https://phytozome-next.jgi.doe.gov/).

To identify members of the *GmftsH* gene family, a combination of methods was employed. Initially, the *Arabidopsis thaliana* ftsH family protein sequence was acquired from the online TAIR database (https://www.arabidopsis.org/) and subjected to BLASTp alignment. From the Pfam database (https://www.ebi.ac.uk/interpro/) [30], the *ftsH* gene family (PF06480; https://www.ebi.ac.uk/interpro/entry/pfam/PF06480/) was downloaded, and a hidden Markov model (HMM) using HMMER3.0 (http://hmmer.org/) with default parameters was applied to screen the *GmftsH* genes from soybean genome data. An e-value of le^− 5^ was set as the threshold for both HMMER and BLASTp searches, and the results were integrated to identify members of the *ftsH* family.

The molecular and chemical characteristics of *GmftsH* family members, including molecular weight (MW), isoelectric point (pl), and protein amino acid count, were analyzed using online ExPASy analysis software (https://web.expasy.org/protparam/). Prediction of subcellular localization was performed using Cell-Ploc2.0 (http://www.csbio.sjtu.edu.cn/bioinf/plant/).

### Phylogenetic analysis

To align the ftsH protein sequences of soybean, rice, corn, and *Arabidopsis thaliana*, we employed the default parameters of ClustalW [31].The phylogenetic tree was constructed using the MEGA11 software [32] with the Maximum Likelihood method (ML) and default settings (BootStrap 1000). After we finished the analysis, we saved the file in NWK format and then used Chiplot (https://www.chiplot.online/) to enhance its appearance.

### Gene structure and chromosomal location of GmftsH genes

For the prediction of conserved motifs in this study, the protein sequence of the GmftsH family was submitted to Multiple Expectation maximizations for Motif Elicitation (MEME; http://meme-suite.org/tools/meme). The number of predicted conserved motifs was set to 10, with a length of 650 aa. The sequence was also submitted to the Conserved Domains Database (CDD; https://www.ncbi.nlm.nih.gov/Structure/cdd/cdd.shtml) to predict the structural domain information, with the e-value set to le-5. The promoter sequence was obtained by extracting the 2000 bp sequence upstream of the start codon for each gene in the *GmftsH* gene family. To predict cis-acting components in the *GmftsH* promoter, PlantCARE (http://bioinformatics.psb.ugent.be/webtools/plantcare/html/) was used for modification. For visualization of the promoter cis-acting elements, ggplot2 package (https://github.com/tidyverse/ggplot2) was utilized. Finally, a diagram of the *GmftsH* family genes structure combination and chromosome distribution was visualized using TBtools software [33].

### Collinearity analysis

MCScanX software [34] was used to analyze the duplication type of *GmftsH* gene family and the collinearity within its genome. Pairwise comparisons of genome-wide protein sequences of the four species were performed using BLAST, set the e-value to le-5, submitted the comparison results to MCScanX for inter-species and intra-species collinearity analysis, extracted the location of the *ftsH* gene from the results of collinear blocks, and counted the number of collinear gene pairs in order to further analyze the differentiation of repeated genes. Circos software (http://circos.ca/citations/) [35] was used to draw the diagram.

### Expression and GO enrichment analysis of GmftsH

The expression profile data of different parts of soybean seedlings at the V2 stage were downloaded from the Plant Public RNA-Seq Database (PPRD; https://plantrnadb.com/), and used TBtools to draw a heatmap. Subsequently, g:Profiler (https://biit.cs.ut.ee/gprofiler/gost) was utilized to conduct an enrichment analysis of the identified 18 genes from the *GmftsH* family, allowing for an initial prediction of their gene functions.

### Plant materials and stress treatment

Jilin Agricultural University’s Biotechnology Center supplied the JiNong74 variety of soybeans. Plump and well-developed soybean seeds were selected, sown in soil, and placed in an artificial culture room for cultivation at a temperature of 25 °C with a light/dark cycle of 16/8 hours. Soybean seedlings at the V2 stage were subjected to stress conditions including temperature of 4 °C, 20% PEG-6000, and 100 mmol/L NaCl. The leaves were gathered at various time points (0, 2, 4, 6, and 8 h) following treatment and promptly frozen in liquid nitrogen for future utilization in RNA extraction. Each experiment was replicated 3 times.

### Quantitative real-time PCR (qRT-PCR)

The expression of the target genes was measured under different stress conditions. Total RNA was extracted from soybean roots, stems, and leaves at the V2 stage using RNAiso Plus (Takara Bio, Kyoto, Japan).The concentration of RNA was measured using a NanoDrop 2000 spectrophotometer (Thermo Fisher, Waltham, MA, USA). The RNA was then reverse transcribed into cDNA using an All-in-One First-Strand cDNA Synthesis Kit (GeneCopoeia Inc., USA) with a dilution factor of 5. The reverse transcription reaction was carried out at 95 °C for 30 s, 95 °C for 10 s, and 60 °C for 30 s, for a total of 40 cycles. The soybean *β-actin* gene (GenBank accession number: NM_001252731.2) was chosen as the internal reference gene. Primers were designed online (https://sg.IDTDNA.com/pages/tools; received on September 10, 2022. See Table S1). The specificity of primers was checked using the NCBI BLAST tool (https://www.ncbi.nlm.nih.gov/tools/primer-blast/). For the experimental evaluation, 3 biological replicates were selected. An RNA concentration of 412.5 ng/µL was used. To eliminate the potential for DNA contamination, DNase I (70µL Reaction + 10µL DNase I) buffer was added, and then the RNA was used for qRT-PCR experiments. qRT-PCR analysis was performed using Mx3000P (Agilent Technologies, Lexington, MA, USA). The expression levels were calculated using the 2^-ΔΔCt^ formula [36]. GraphPad Prism 9.5.0 software (https://www.graphpad-prism.cn/) was employed to generate histograms.

### GmftsH7 subcellular localization analysis

RNA was extracted from the tender leaves of soybeans, was reverse-transcribed into cDNA, and was used as a PCR template to clone the full-length coding region of *GmftsH7*. *Nicotiana benthamiana* leaves aged 6 ~ 8 weeks were selected for injection. Seamless cloning technology was used to construct the pCAMBIA1302-GmftsH7-GFP vector, which was then transferred into *Escherichia coli*, and the recombinant plasmid was extracted. The pCAMBIA1302-GFP and pCAMBIA1302-GmftsH7-GFP plasmids were transferred into *Agrobacterium tumefaciens* strain GAH105 recipient cells. Both types of bacterial cultures were propagated and were resuspended in infiltration buffer. After incubation at 28℃ for 3 h, a 1 mL syringe was used to gently pierce a small opening on the underside of the tobacco leaf (being careful not to puncture through) and then, with the needle removed, the syringe was used to draw up the bacterial solution and inject it into the leaf through the wound (supporting the front side of the leaf with fingers to allow the bacterial solution to permeate from the back). The wet areas on the tobacco leaf were marked with a marker. 48 h later, gene expression and localization were observed under a laser confocal microscope.

## Results

### Identification of GmftsH family

In this study, we identified 18 *GmftsH* gene family members (Table 1), analyzed their physical and chemical properties, and predicted their subcellular localization. GmftsH4 is a protein comprising 320 amino acids (aa), indicating a relatively shorter structure, whereas GmftsH15 is a notably longer protein, consisting of 848 aa. The protein has a molecular weight range of 35.85 Da (GmftsH4) to 99.55 Da (GmftH15), and the PI range is 5.41 (GmftsH12) to 10.05 (GmftsH16). Subcellular localization prediction results show that many members of the *GmftsH* gene family are expressed in chloroplasts and nucleus (*GmftsH5*, *GmftsH6*, *GmftsH9*, *GmftsH13*, *GmftsH15*, *GmftsH16*, and *GmftsH18*), and some are only expressed in chloroplasts (*GmftsH2*, *GmftsH3*, *GmftsH8*, *GmftsH10*, *GmftsH11*, *GmftsH12*, *GmftsH14*, and *GmftsH17*). On the other hand, *GmftsH1* gene is only expressed in the nucleus, and *GmftsH7* gene is expressed in the cell membrane.

**Table 1:** List of 18 *GmftsH* gene family members identified in this study

Gene	Gene ID	Chr	Start	End	Forward/ reverse strand	Length	MolWt	pI	Subcellular localization
*Glyma.04G213800*	*GmftsH1*	Gm04	47,367,231	47,376,286	+	844	94795.31	5.66	Nucleus.
*Glyma.04G019100*	*GmftsH2*	Gm04	1,495,640	1,499,745	-	695	74160.56	6.03	Chloroplast.
*Glyma.02G225300*	*GmftsH3*	Gm02	43,089,646	43,098,031	+	804	86930.02	8.73	Chloroplast
*Glyma.12G061500*	*GmftsH4*	Gm12	4,475,583	4,478,866	+	320	35864.88	9.06	Cell membrane. Cell wall. Chloroplast.
*Glyma.12G061200*	*GmftsH5*	Gm12	4,434,099	4,440,167	+	811	89316.76	8.1	Chloroplast. Nucleus.
*Glyma.12G061400*	*GmftsH6*	Gm12	4,456,032	4,466,507	+	807	88528.65	7.17	Chloroplast. Nucleus.
*Glyma.08G086600*	*GmftsH7*	Gm08	6,554,799	6,557,920	-	697	74980.52	5.7	Cytomembrane.
*Glyma.15G158900*	*GmftsH8*	Gm15	13,376,352	13,381,817	+	691	74096.77	6.04	Chloroplast.
*Glyma.11G137700*	*GmftsH9*	Gm11	10,453,054	10,458,893	+	811	89324.67	8.63	Chloroplast. Nucleus.
*Glyma.09G052600*	*GmftsH10*	Gm09	4,575,415	4,579,489	+	696	74631.55	6.04	Chloroplast.
*Glyma.13G049800*	*GmftsH11*	Gm13	13,638,595	13,644,343	-	639	70631.46	9.84	Chloroplast.
*Glyma.05G132000*	*GmftsH12*	Gm05	32,540,663	32,544,287	-	696	74816.31	5.41	Chloroplast.
*Glyma.14G192100*	*GmftsH13*	Gm14	46,553,229	46,561,347	+	813	88164.59	9.09	Chloroplast. Nucleus.
*Glyma.06G019400*	*GmftsH14*	Gm06	1,461,094	1,465,242	-	697	74393.95	6.09	Chloroplast.
*Glyma.06G152500*	*GmftsH15*	Gm06	12,379,423	12,388,055	-	848	95554.22	5.97	Chloroplast. Nucleus.
*Glyma.19G040200*	*GmftsH16*	Gm19	5,780,996	5,786,677	+	632	69692.62	10.05	Chloroplast. Nucleus.
*Glyma.18G259700*	*GmftsH17*	Gm18	54,853,317	54,857,662	-	679	73349.2	6.04	Chloroplast.
*Glyma.18G065600*	*GmftsH18*	Gm18	5,942,975	5,951,508	+	793	86240.29	8.45	Chloroplast. Nucleus.

**Fig. 1:**
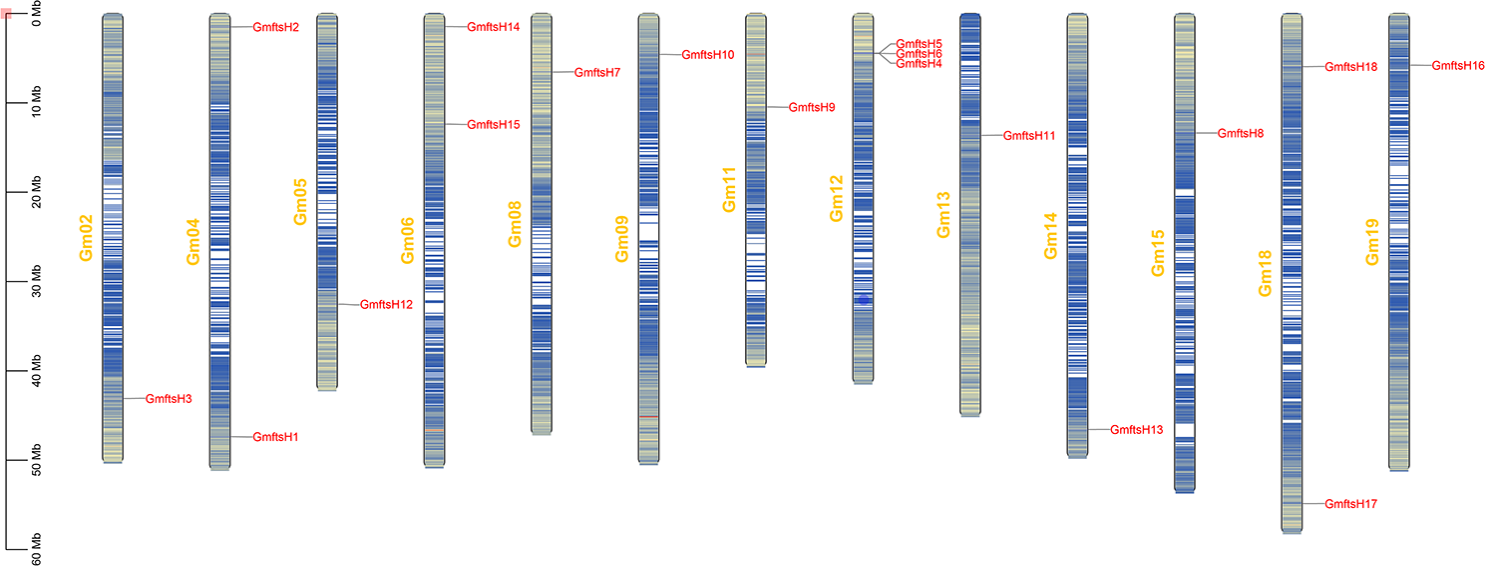
Chromosome distribution of *GmftsH* gene family. Sliding window size set to 100 kb, with yellow to blue indicating gene density from high to low

The 18 members of the *GmftsH* gene family are unevenly distributed on 13 chromosomes (Fig. 1). Among them, the *GmftsH* genes on chromosomes Gm06, Gm08, Gm09, Gm11, Gm12, Gm13, Gm15, and Gm19 are located at the front end of the chromosome, and those on chromosomes Gm02, Gm05, and Gm14 are located at the tail end of the chromosome. The positions of *GmftsH4*, *GmftsH5*, and *GmftsH6* on chromosome Gm12 are close, similar to the proximity of *GmftsH14* and *GmftsH15* on chromosome Gm06.

### Phylogenetic analysis of GmftsH gene family

**Fig. 2:**
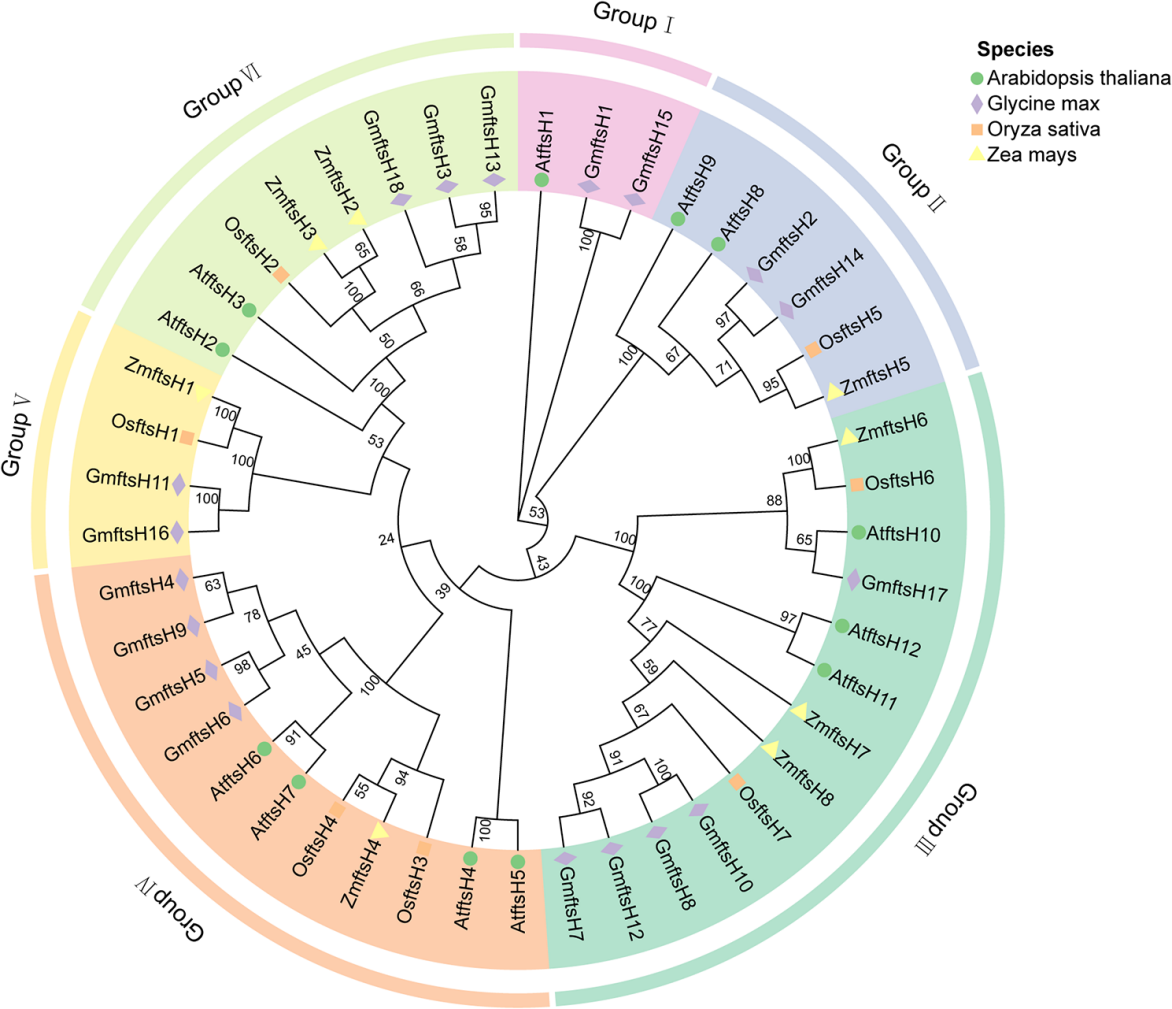
Phylogenetic tree of ftsH proteins from soybean, Arabidopsis, maize, and rice. Groups I–VI represent subgroups I–VI.

A comparison of the *ftsH* genes in rice, maize, *Arabidopsis thaliana*, and soybean reveals homology among them. This phylogenetic analysis categorizes the 45 *ftsH* genes into six distinct subgroups, with the 18 *GmftsH* genes from soybean being distributed across various subfamilies. Group I is comprised exclusively of *ftsH* gene members from dicotyledonous plants. The largest of these subgroups is Group III, encompassing five members - *GmftsH7*, *GmftsH8*, *GmftsH10*, *GmftsH12*, and *GmftsH17*. Additionally, Group IV includes four soybean *ftsH* genes, which exhibit a close phylogenetic relationship with the *ftsH* genes found in *Arabidopsis thaliana*(Fig. 2). This pronounced homology between the Arabidopsis and soybean *ftsH* genes, in contrast to those in rice and maize, could be attributed to their commonality as dicotyledonous plants. Integrating this with subcellular localization predictions, it appears that genes expressed within similar cellular compartments are more likely to be closely related, potentially indicative of parallel functional roles. Consequently, this suggests a likelihood that GmftsH and AtftsH proteins may fulfill analogous biological functions within the cellular framework.

### Structure and motif composition of GmftsH gene family

**Fig. 3:**
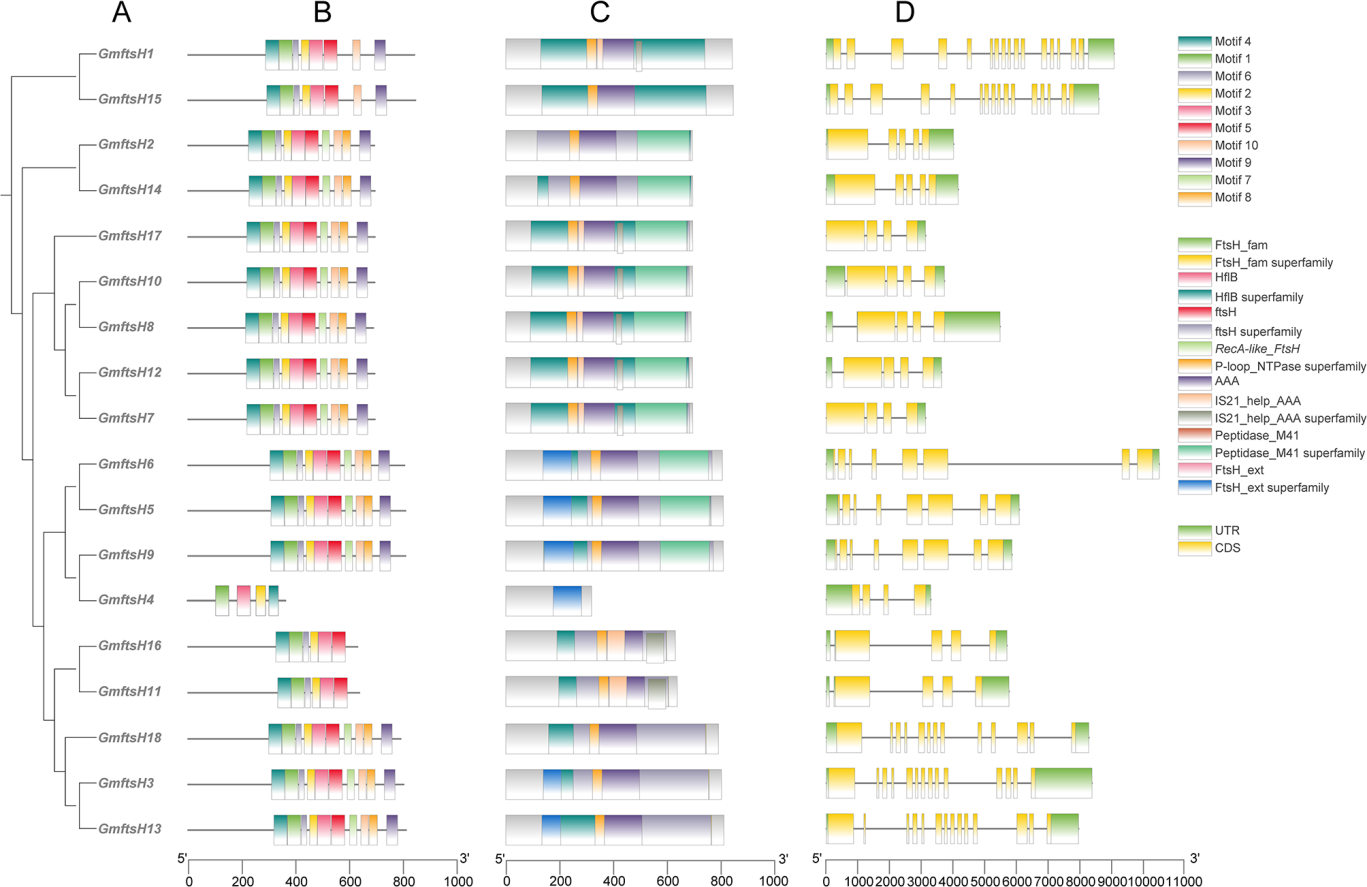
Phylogenetic tree, gene structure, protein conserved motifs, and conserved domain analysis of *GmftsH* gene family. (**A**) Phylogenetic tree constructed by maximum likelihood method (**B**) Motif analysis of protein encoded by *GmftsH* genes. (**C**) Conserved domain of *GmftsH* genes. (**D**) *GmftsH* genes structure

All *GmftsH* genes contain multiple exons and introns (Fig. 3D). The number and length of introns and exons of genes located on the same evolutionary tree branch are roughly similar. The number of exons in the genes ranges from 16 to 4, with *GmftsH10*, *GmftsH8*, *GmftsH12*, *GmftsH7*, *GmftsH4*, and *GmftsH16* each comprising four exons. The presence of ftsH-related conserved domains is a common feature among all *GmftsH* genes, which is characteristic of the ftsH protein family. To determine the composition and number of these conserved motifs in the *GmftsH* gene family (Fig. 3A, C), we utilized online MEME software. This analysis identified a total of 10 conserved motifs, labeled Motif 1 to Motif 10 (Fig. 3B). All *GmftsH* genes share these conserved motifs, whereas *GmftsH4*, *GmftsH11*, and *GmftsH16* lack Motifs 8 and 9. The diversity of the soybean *ftsH* gene family is related to the distribution and structure of conserved motifs, and it was observed that genes on the same branch possess the same number and type of motifs.

**Fig. 4:**
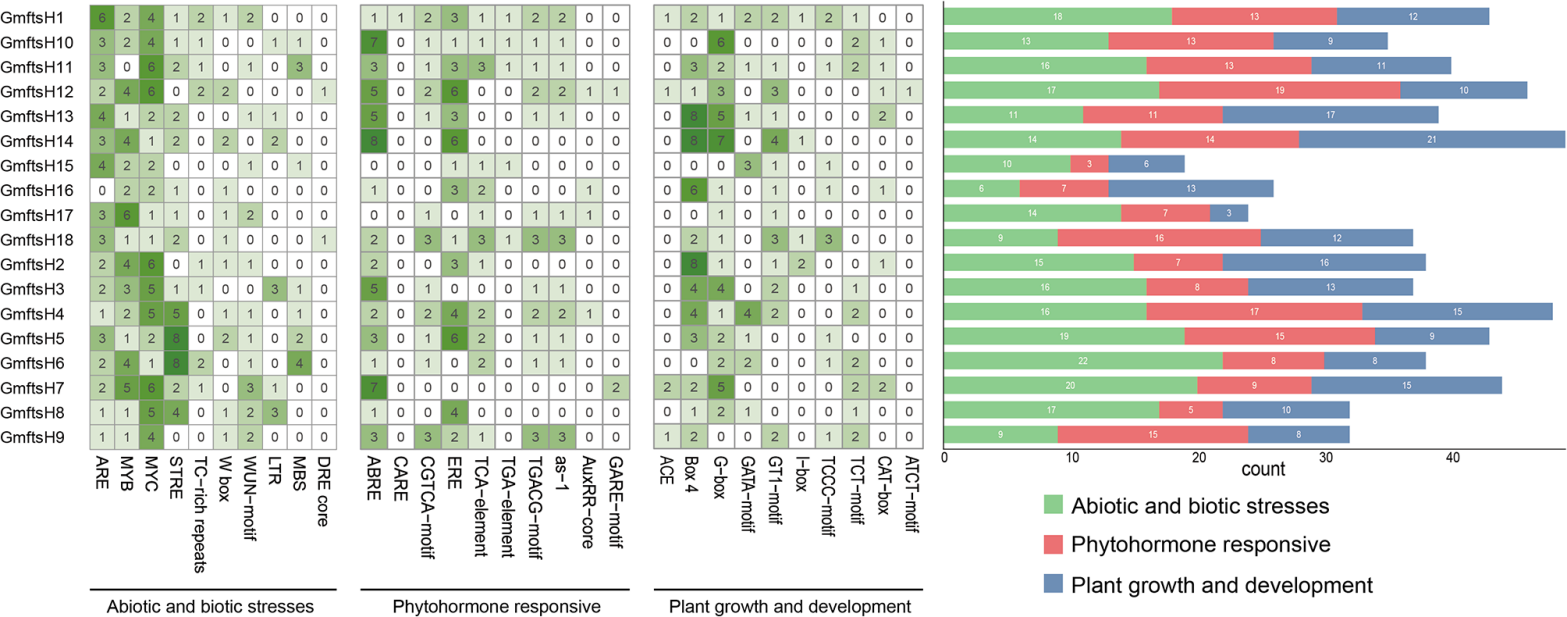
Analysis of cis-acting elements in promoter region of *GmftsH* gene family

In order to investigate gene expression levels, the promoter regions located 2000 bp upstream of 18 genes in the *GmftsH* gene family were analyzed. The analysis results (Fig. 4) indicate that the upstream promoters of the 18 *GmftsH* genes contain multiple elements associated with abiotic and biotic stress, plant hormone response, and plant growth and development. Within these, the cis-acting elements related to abiotic and biotic stress were the most abundant, similar in number to those associated with plant hormone response and plant growth and development. Cis-acting elements such as MYB, ABRE, and G-box, which respond to biotic stress, have been identified in different experiments. *GmftsH14*, *GmftsH4*, and *GmftsH6* contain drought-inducible, low-temperature-inducible, and abscisic acid response elements, as well as MYB binding sites, so these three genes are likely to play important roles in drought and low-temperature stress.

### Intraspecific collinearity analysis of GmftsH gene family

**Fig. 5:**
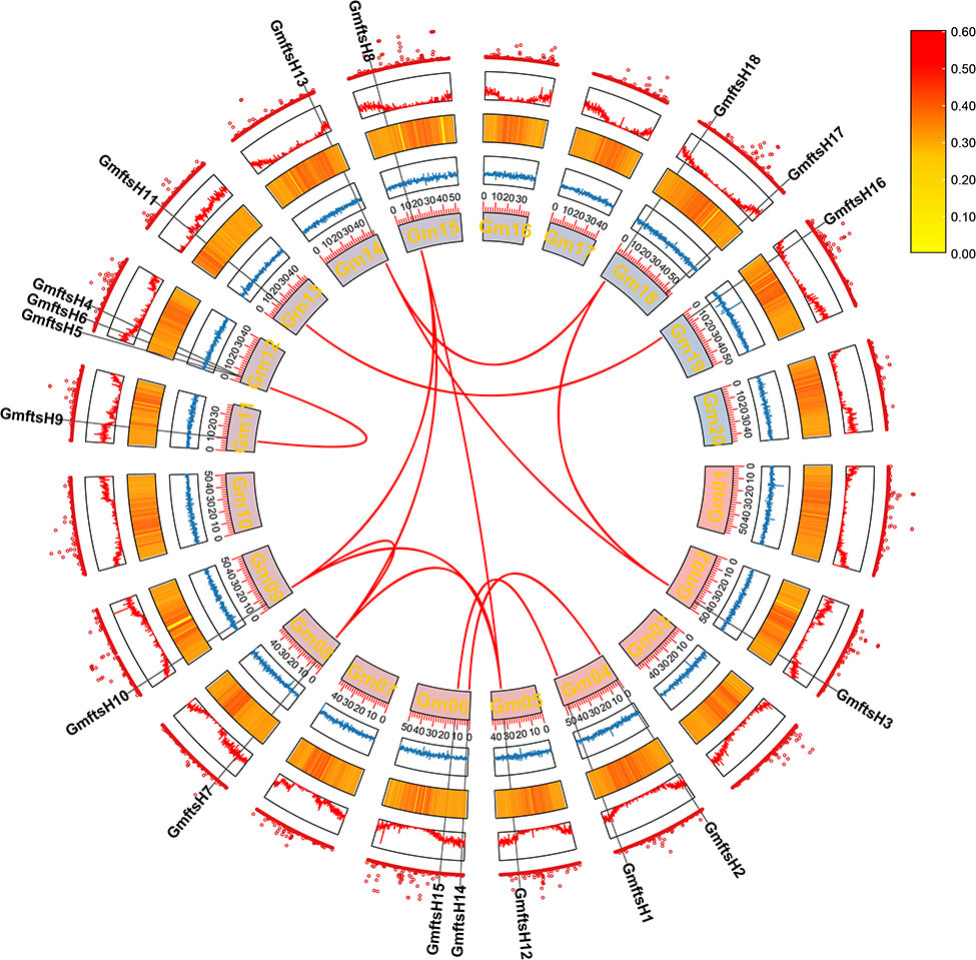
Collinearity analysis of *GmftsH* gene family. Repeated gene pair segments are connected by red lines, with red and yellow corresponding to high- and low-density values

During plant evolution, gene duplication and retention of existing genes allow plant genomes to expand. As can be seen from Fig. 5, a total of 13 pairs of collinear soybean *ftsH* genes were detected across the 20 chromosomes of soybean, with the highest number of collinear gene pairs found on chromosomes 5, 8, 9, and 15. Among the 20 chromosomes (Gm1-Gm20), 18 *GmftsH* genes are unevenly distributed on the remaining chromosomes. Three *GmftsH* genes are located on chromosome Gm12; two on chromosomes Gm6, Gm4, and Gm18; and one each on chromosomes Gm2, Gm5, Gm8, Gm9, Gm11, Gm13, Gm14, Gm15, and Gm19. In the Circos diagram (Figs. 5), 13 gene pairs with segmental duplications are connected by red curves. From the gene position analysis, it can be seen that *GmftsH6* is produced by tandem duplication, and no gene duplication event occurs in the *GmftsH17* gene, indicating that in *GmftsH* gene amplification, the fragment replication ratio is greater than the tandem replication ratio. The expansion of the *GmftsH* gene family is associated with events of tandem and segmental duplication.

### Interspecific collinearity analysis of GmftsH gene family

**Fig. 6:**
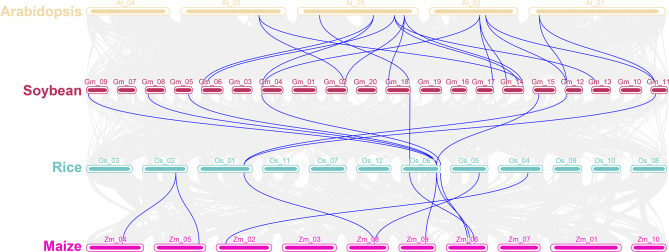
Collinearity analysis of *ftsH* gene families in soybean, corn, rice, and *Arabidopsis thaliana*. Gray line indicates all syntenic blocks in genome; blue line indicates large fragment duplicated *ftsH* gene pairs

The collinearity relationship between *GmftsH* gene family members and Arabidopsis, rice, and maize *ftsH* genes was further analyzed. There were 18 collinear fragments identified between *Arabidopsis thaliana* and soybean, 8 collinear fragments between rice and soybean, and 9 collinear fragments between rice and maize (Fig. 6). Based on these collinear relationships, a collinear plot was drawn to represent homologous gene pairs of soybean and other species, connected by blue lines in Fig. 6. Soybean and Arabidopsis have the most collinear fragments, located on chromosomes 1, 2, 3, and 5 of Arabidopsis. The abundance of homologous *ftsH* gene pairs indicates high homology between soybean and Arabidopsis.

### GO enrichment analysis of GmftsH gene family

**Fig. 7:**
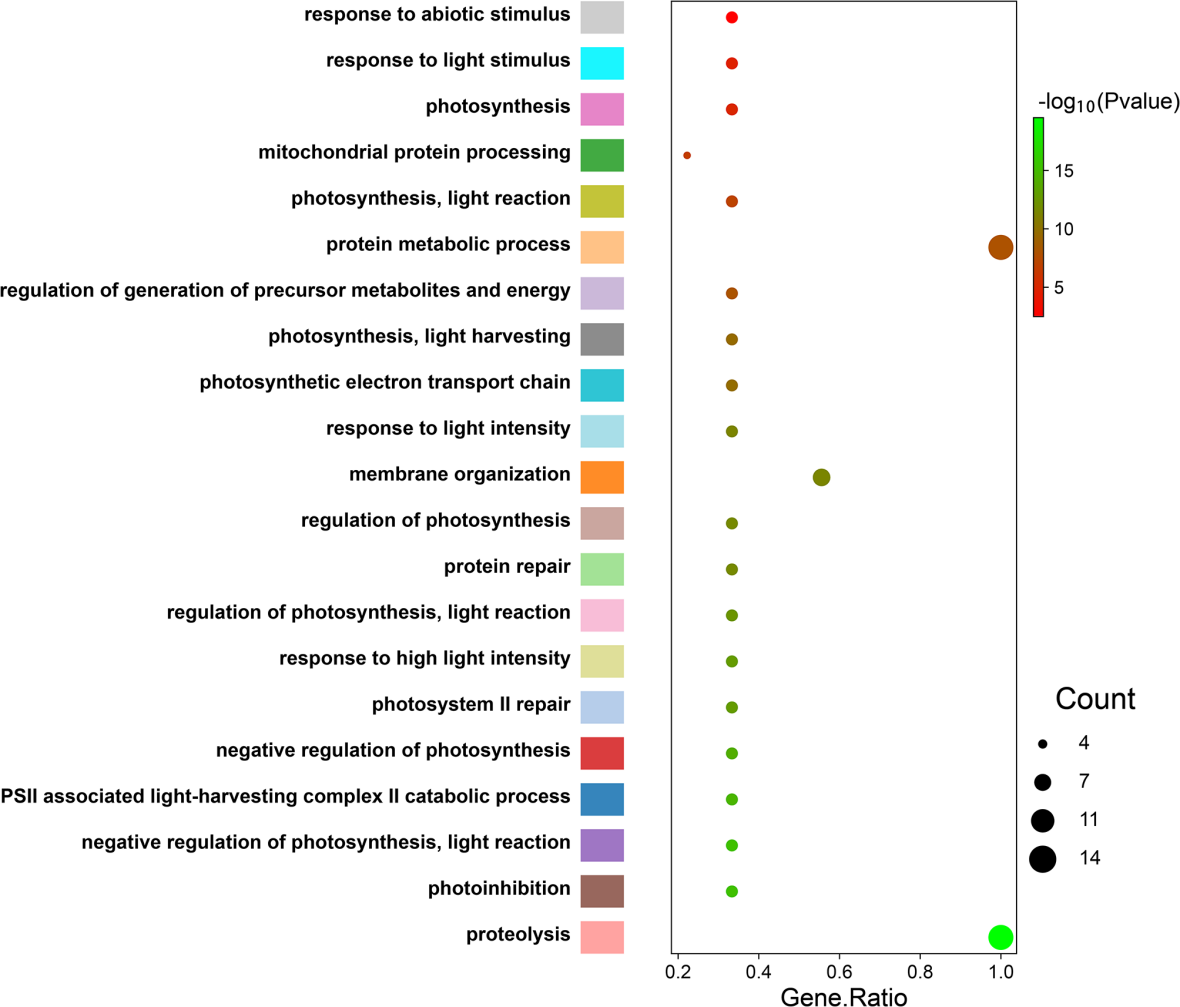
GO enrichment analysis of *GmftsH* gene family members

The results show that the *GmftsH* genes are mainly involved in the response to abiotic and light stimuli, mitochondrial protein processing, regulation of light-responsive protein metabolism, and energy generation. The *GmftsH* genes exhibit diverse molecular functions as well, including ATP-dependent peptidase activity, catalytic enzyme activity, hydrolase activity, and metalloprotease activity (Fig. 7).

### Analysis of tissue expression pattern of GmftsH gene family

**Fig. 8:**
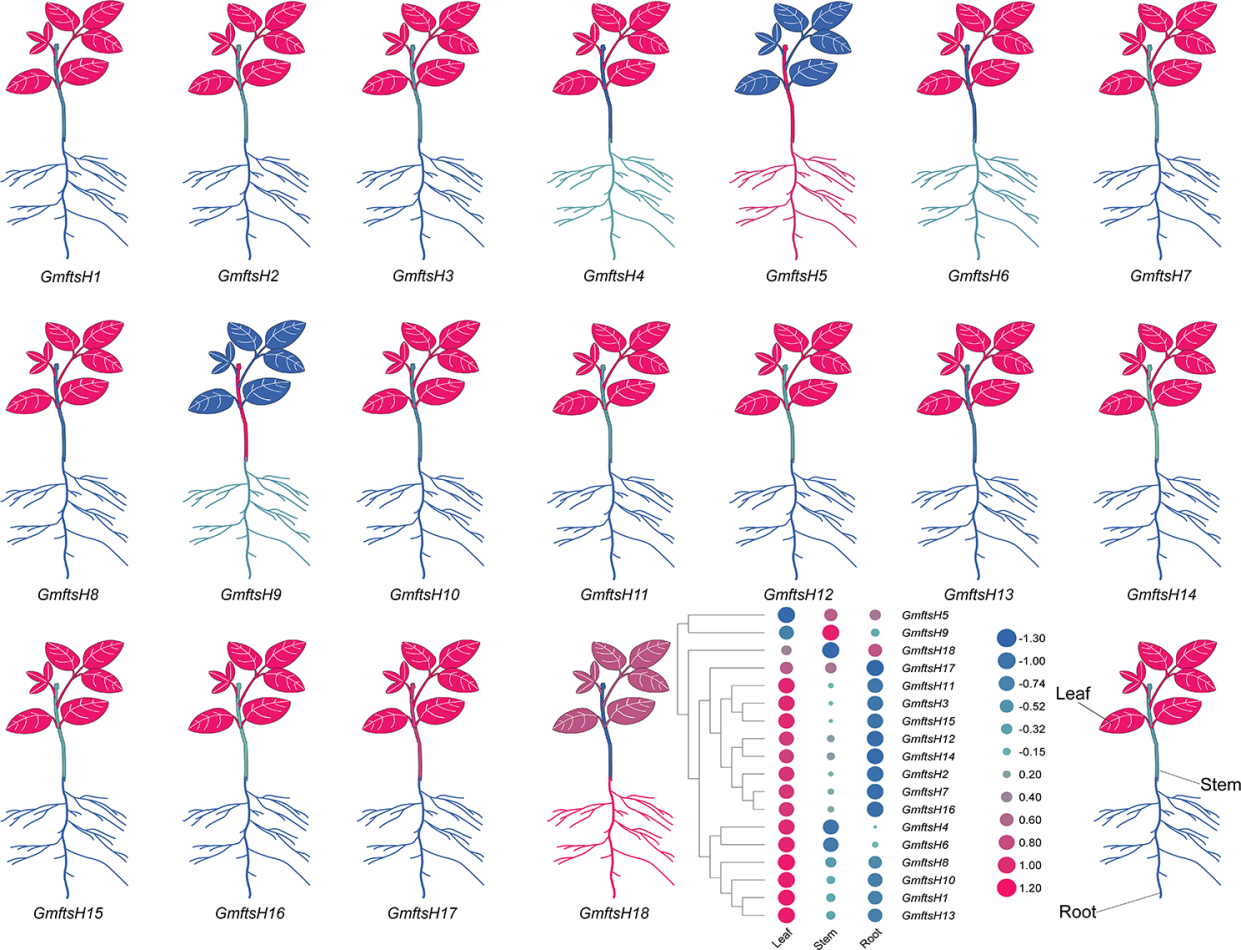
Expression patterns of *GmftsH* gene family members were analyzed in three tissue types. Expression level in terms of Log_2_FPKM is indicated in blue for low-abundance transcripts and pink for high-abundance transcripts

It can be seen from Fig. 8 that *GmftsH* genes have different expression patterns. Most genes are expressed in three parts: roots, stems, and leaves. *GmftsH9* is highly expressed in stems. *GmftsH18* and *GmftsH5* are highly expressed in roots, indicating their essential roles in root development. Except for *GmftsH9* and *GmftsH5*, the remaining 16 genes are highly expressed in leaves and may play a role in the growth and photosynthesis of leaves (Fig. 8). In this study, the 18 *GmftsH* genes were expressed in at least one tissue, indicating that the *GmftsH* gene family functions in different soybean tissues and organs.

**Fig. 9:**
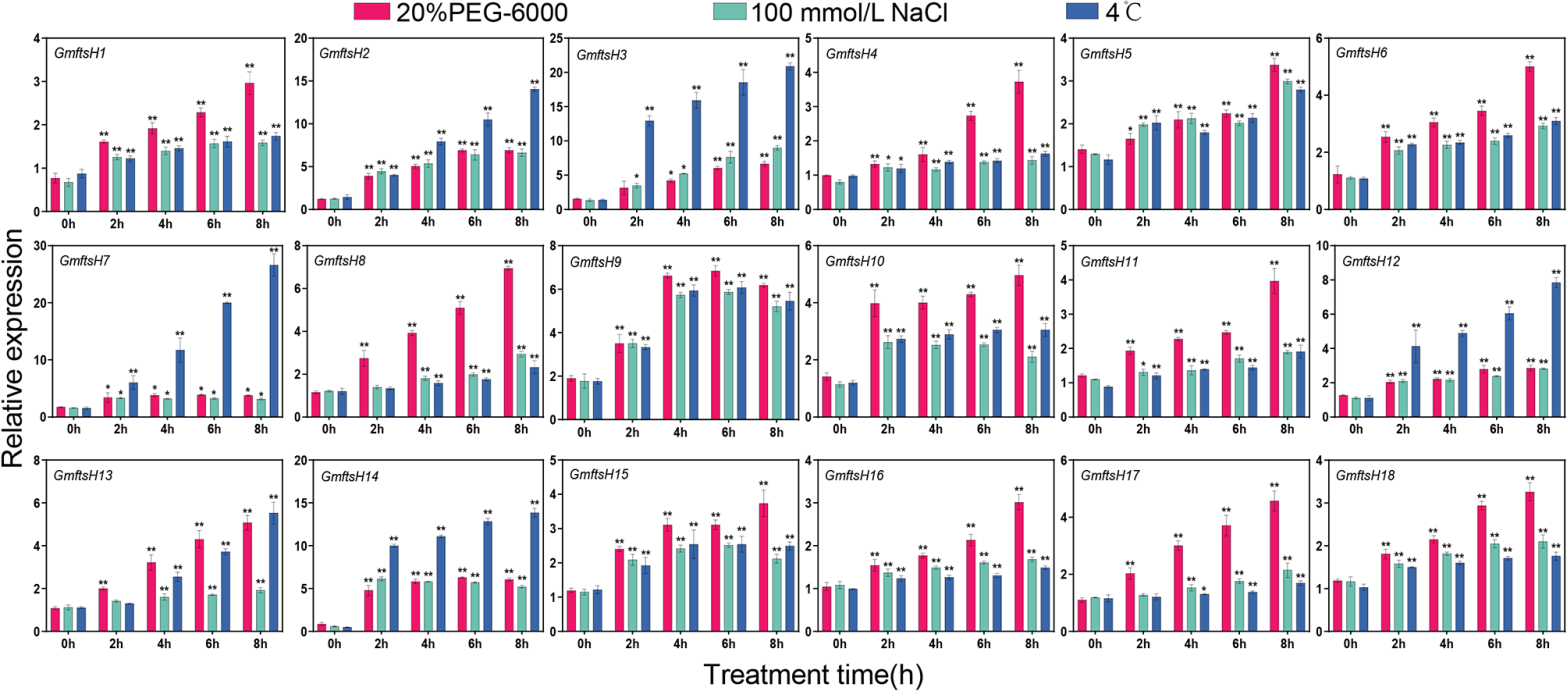
Analysis of expression levels of *GmftsH* gene family members under different stresses. T-test was used; asterisks indicate statistically significant differences (* *p* < 0.05; ** *p* < 0.01)

We analyzed gene expression under different stress conditions to investigate the potential involvement of *GmftsH* genes. The expression of 18 *GmftsH* genes in roots, stems, and leaves was evaluated using qRT-PCR. The genes exhibited distinct expression patterns under three abiotic stress conditions: 20% PEG-6000, 100 mmol/L NaCl, and 4 °C. The majority of genes showed significant changes in expression during the early stages of the response (Fig. 9). For example, under 20% PEG-6000 treatment, the expression of *GmftsH8 i*n leaves increased significantly, reaching a peak at 8 h of treatment; both *GmftsH8* and *GmftsH14* had increased expression with different extended treatment times, but *GmftsH8* The expression level was slightly lower than that of *GmftsH14*. The expression level of *GmftsH7* increased by about three times compared with the expression level at 2 h under 4 °C treatment for 8 h.

### Subcellular localization

**Fig. 10:**
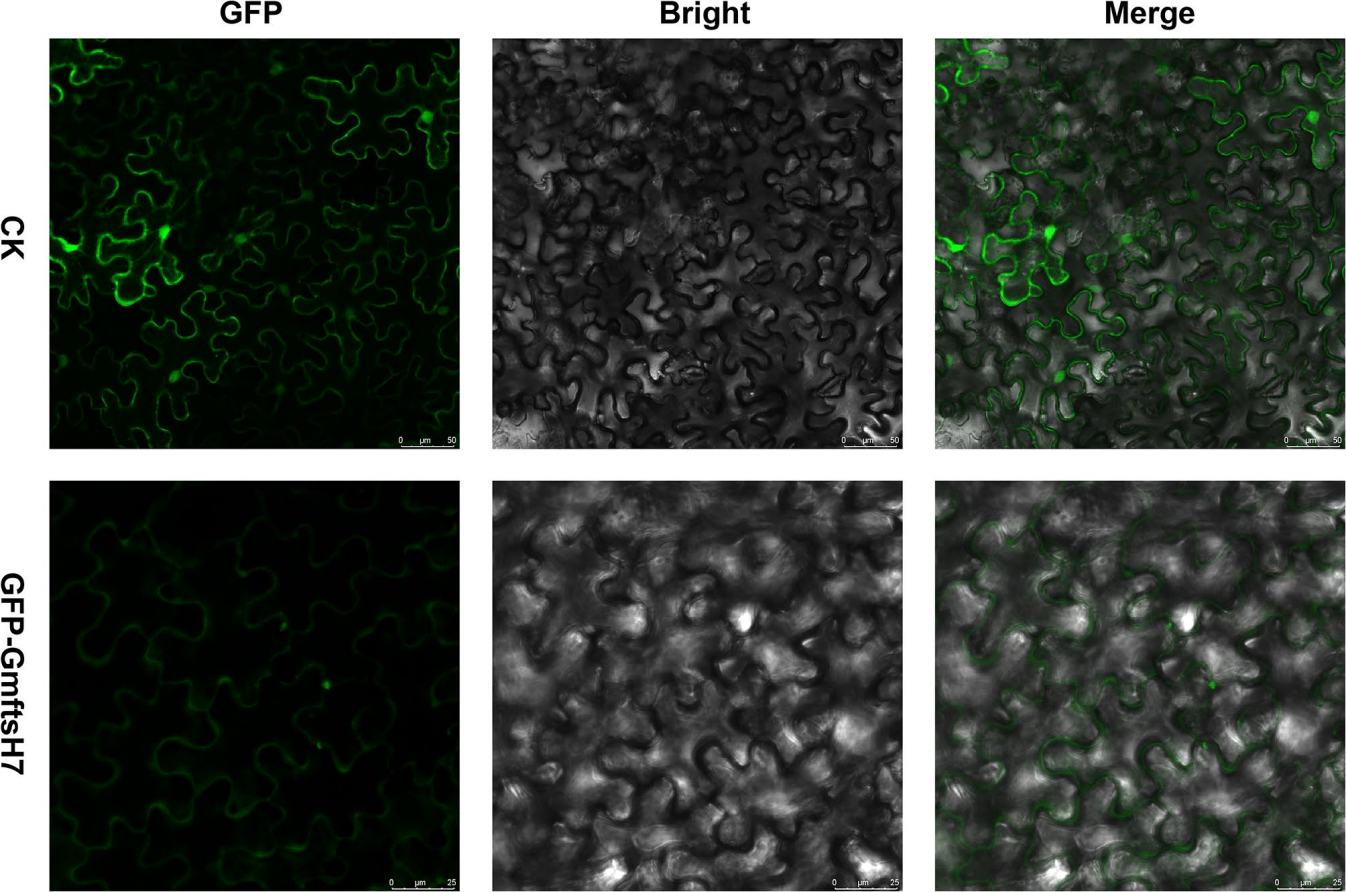
Subcellular localization results of *GmftsH7* gene. Note CK, pCAMBIA1302-GFP; GFP, green excitation light state; Bright, bright field; Merge, superposition state

The functional analysis of genes relies strongly on understanding where the expression products of genes are located within cells. In order to determine the expected subcellular location of *GmftsH7*, we analyzed the temporary expression of pCAMBIA1302-GFP and pCAMBIA1302-GmftsH7-GFP in N. benthamiana leaves. A green fluorescence signal of non-linked GFP could be observed in both the nucleus and cytoplasm, as shown in Fig. 10. However, the green fluorescence signal of GmftsH7-GFP was solely localized to the cell membrane. This confirms that *GmftsH7* is indeed situated on the cell membrane.

## Discussion

The *ftsH* genes is widely found in prokaryotes and eukaryotes, and plays important roles in plant growth and development and resistance to abiotic stress [37–39]. The *ftsH* gene families in *Arabidopsis thaliana*, maize, and rice have previously been identified and analyzed, but those in soybean have not been reported. In this study, based on the similarity of ftsH protein with that in *Arabidopsis thaliana* and the existence of the ftsH_ext domain, we performed genome-wide identification and a systematic analysis of Soybean *ftsH* gene family and identified a total of 18 genes, which were more similar than the *ftsH* genes in Arabidopsis, corn, rice, and tobacco [40–42]. This suggests that the *ftsH* genes may have experienced sustained, family-specific amplification throughout evolution [43, 44].

According to the phylogenetic relationship, the *ftsH* gene family in soybean, rice, maize, and Arabidopsis is divided into six subfamilies, with a greater number of soybean *ftsH* genes being distributed in Group III. Soybean is closely related to *Arabidopsis thaliana* (Fig. 2), and its gene structure, subcellular localization, conserved motifs, evolutionary relationships, and expression patterns were comprehensively analyzed. By studying the gene structure, we found a substantial presence of introns in soybean *ftsH* genes, numbering between 3 and 15. Genes on the same evolutionary branch have similar intron–exon composition and conserved domains (Fig. 3), suggesting that these genes may have similar functions in plants. *GmftsH* is similar in structure and conserved domain to *ftsH* gene members reported in tomato, pepper, and potato [45–47]. Typical ftsH features include an N-terminal transmembrane structure consisting of one or two transmembrane helices and a highly conserved AAA + ATPase domain [48, 49], which has been shown to function in the hydrolysis and transformation of ATP [50].

The results of prediction analysis of promoter cis-acting elements of *GmftsH* genes (Fig. 4) partially coincided with the GO enrichment results (Fig. 7). This gene family may be involved in abiotic stress response, photosynthesis, and mitochondrial protein processing in plants, suggesting that *ftsH* genes may be closely related to photosynthesis. This has been confirmed in organisms that perform photosynthesis, such as cyanobacteria and Arabidopsis. For example, plants with reduced *FTSH12* gene expression will have pale leaves and malformed chloroplasts [41, 51]. The collinearity analysis showed that the soybean *ftsH* gene family has 13 gene pairs with segment repeats (Fig. 5), and the most collinear fragments among species were identified in Arabidopsis (Fig. 6).

We analyzed the expression patterns at different tissues of the *GmftsH* gene family genes to better understand their potential biological functions. In this study, most of the 18 *GmftsH* genes were highly expressed in leaves (Fig. 8), which may indicate that they have a similar function to the homologous Arabidopsis *ftsH* gene and play a role in photosynthesis [42]. *GmftsH5* and *GmftsH18* were highly expressed in roots, which may indicate that they have a similar function to the homologous rice *ftsH* genes and play a role in drought stress and root nutrient metabolism [52]. Previous studies found that the tomato *LeftsH6* gene shows strong heat-induced GUS staining in ovary, stigma, anther, and sepal at different developmental stages, as well as pollen grains of mature anthers, and its heat induction is mediated by the interaction between HSEs of *LeftsH6* promoter and heat shock factor [47]. Similarly, it was found that Arabidopsis *FtsH6* can regulate the expression of *HSP21*. A lack of functional FtsH6 protein can promote the accumulation of *HSP21* and increase the thermal memory capacity of Arabidopsis [53].

We also analyzed the gene expression patterns of the *GmftsH* gene family under three abiotic stress conditions (Fig. 9). The results show different expression levels of most *GmftsH* genes under different abiotic stress conditions. For example, the expression levels of *GmftsH4*, *GmftsH8*, and *GmftsH10* were significantly increased under 20% PEG-6000 treatment. Under 100 mmol/LNaCl treatment, the expression level of *GmftsH9* was higher than that of other genes, but the expression level of *GMFTSH9* under induced drought was slightly higher than that under salt stress. At the same time, the expression level of *GmftsH7* in leaves treated at 4 ℃ for 8 h was about three times higher than that at 2 h, and the expression level in leaves reached a very significant level. This is consistent with the results that the *ftsH* gene was induced to be expressed in alfalfa under low-temperature treatment [27]. Overexpression of *ftsH* can improve the heat and salt tolerance of plants, *ftsH* mutants reduce the capacity of biofilm formation [54]. Our research results show that *GmftsH* gene is involved in plant response to abiotic stress, which is consistent with the analysis results of cis-acting elements. It may be that these cis-acting elements are bound by transcription factors under stress and regulate the expression of target genes, thus they play a role in plant resistance to abiotic stress. There are few studies on low-temperature treatment of *GmftsH* genes, and the function of *GmftsH* in soybean in response to low-temperature stress needs to be further verified.

## Conclusions

In this study, a total of 18 *GmftsH* genes were found in the soybean genome, and these genes were unequally distributed on 13 chromosomes. Among them, 17 *GmftsH* genes were generated by segment duplication and *GmftsH6* was generated by tandem duplication, indicating that, in the amplification of *GmftsH* family genes, the proportion of segment duplication is greater than the proportion of tandem duplication. An analysis of the gene transcriptome data of the 18 *GmftsH* genes showed that these genes play a role in plant growth and development. qRT-PCR analysis of soybeans under abiotic stress showed that *GmftsH* family genes may be involved in the abiotic stress response. Among them, *GmftsH7* was significantly upregulated under 4 ℃ treatment; thus, it may play a vital role in cold tolerance. Subcellular localization showed that *GmftsH7* was localized in the cell membrane. The research results will help in analyzing the molecular mechanism of the *ftsH* gene family in the growth and development of soybeans and provide genetic resources for improving soybean stress resistance traits through molecular breeding.

### Electronic supplementary material

Below is the link to the electronic supplementary material.

Supplementary Material 1

## Data Availability

The sequence information for the entire soybean genome was obtained from the Phytozome 13 Web site (https://phytozome-next.jgi.doe.gov/). obtained, which is open to all researchers. Soybean (JiNong 74) seeds provided by the Laboratory of Biotechnology Center of Jilin Agricultural University were used as experimental materials. The datasets supporting the conclusions of this paper are included in this paper and its additional files.
