# Supplementary material for: Genome-wide analysis of filamentous temperature-sensitive H protease (ftsH) gene family in soybean
Source: BMC Genomics. 2024 May 27;25:524. doi: 10.1186/s12864-024-10389-w (PMC11131285; doi:10.1186/s12864-024-10389-w)
Supplement: Supplementary file 1 — Supplementary Material 1 [file 12864_2024_10389_MOESM1_ESM.docx]

| Gene | Gene ID | Forward primer | Reverse primer |
| --- | --- | --- | --- |
| *Glyma.04G213800* | *GmftsH1* | CCCTATTTCCTCACTCTTCACC | TGGTTGGGATTGGTTGAGTC |
| *Glyma.04G019100* | *GmftsH2* | GAATGACGAGAGGGAACAGAC | CAACGCCGAATCAAGAACATC |
| *Glyma.02G225300* | *GmftsH3* | TGCTGGAAAATGAGGTGGAG | CTAACAGGGAATCGATGGAGAAG |
| *Glyma.12G061500* | *GmftsH4* | TTTGTTCTATCCAACGTCGACC | TCTCCTTCTTTGGAGCTTTGC |
| *Glyma.12G061200* | *GmftsH5* | GCTTATCGAGGAACACAAGG | CGTTCCTCTGTCAGCAAATTG |
| *Glyma.12G061400* | *GmftsH6* | GCGTGAATGGGTGAACAAAG | GCCCAAAACTCGAAGCAAG |
| *Glyma.08G086600* | *GmftsH7* | GCTGATCTTGCGAATCTGTTG | TTGCTCTTCCCATCTGTCATC |
| *Glyma.15G158900* | *GmftsH8* | GACAGATGGAAAGAGCAAAAGC | AATGAACCAAGTAAGACCCCG |
| *Glyma.11G137700* | *GmftsH9* | ACTCCCCGCAACCAAATAG | TCCCCTCAAAAGACTCAACAC |
| *Glyma.09G052600* | *GmftsH10* | GACAGATGGAAAGAGCAAAAGC | AACCAAGTAAGACCACGAGC |
| *Glyma.13G049800* | *GmftsH11* | CTGCCCCTCAATCTGTACTAAG | ATTCGGTCTCTGCTTCCTTG |
| *Glyma.05G132000* | *GmftsH12* | AAAGAGGGACTGGAATTGGTG | AGGCAGAGTCAAGAATGTCAG |
| *Glyma.14G192100* | *GmftsH13* | CTACCTCAATTCCCACACCTG | TCAGCCTCCACAAATCCAC |
| *Glyma.06G019400* | *GmftsH14* | CAACCCTCATCCCCTTTCTC | TTGCTGAACCTGACTCGC |
| *Glyma.06G152500* | *GmftsH15* | AATTTCCCCACTCTTCACCTC | GGCGTCTTGTTCTTGTTCTTGTGTG |
| *Glyma.19G040200* | *GmftsH16* | CTGGAACTGGGAAGACACTAC | GCTGCATTGAAAAGGTCTCTG |
| *Glyma.18G259700* | *GmftsH17* | GCCCTATGTTTCTCAGTCTCC | GTTGGCTTTTGTGAGGTGTG |
| *Glyma.18G065600* | *GmftsH18* | CCATTATCCGAGCCAATCCTAC | CTCTTCACCCTCAACTTTCTCC |
| *β-actin* | *β-actin* | CTGAGGTTCTATTCCAGCCATCC | CCACCACTGAGGACAACATTACC |

**TableS1.** Primer list for qRT-PCR assay of *GmftsH* family genes.
